# Protective Effects of *Ophiocordyceps lanpingensis* on Glycerol-Induced Acute Renal Failure in Mice

**DOI:** 10.1155/2017/2012585

**Published:** 2017-10-12

**Authors:** Yanyan Zhang, Yaxi Du, Hong Yu, Yongchun Zhou, Feng Ge

**Affiliations:** ^1^Faculty of Life Science and Technology, Kunming University of Science and Technology, Kunming, Yunnan 650500, China; ^2^Tumor Research Institute of Yunnan Province, The Third Affiliated Hospital of Kunming Medical University (Yunnan Tumor Hospital), Kunming, Yunnan 650118, China; ^3^School of Life Sciences, Yunnan University, Kunming, Yunnan 650500, China; ^4^The Research Center of Cordyceps Development and Utilization of Kunming, Kunming, Yunnan 650100, China

## Abstract

**Objective:**

Oxidative stress and immune response are associated with acute renal failure (ARF). *Ophiocordyceps lanpingensis* (OL) might be an antioxidant and immunopotentiator. In this study, we explored the protective effects of OL on glycerol-induced ARF.

**Methods:**

Male mice were randomly divided into four groups, specifically, glycerol-induced ARF model group, low-dose OL-treated group (1.0 g/kg/d), high-dose OL-treated group (2.0 g/kg/d), and control group. Renal conditions were evaluated using kidney index, serum creatinine (Cr), blood urea nitrogen (BUN), and histological analysis. Rhabdomyolysis was monitored using creatine kinase (CK) level. Oxidative stress was determined using kidney tissue glutathione (GSH), malondialdehyde (MDA), and superoxide dismutase (SOD) levels. Immune status was evaluated using immune organ indices and immunoglobulin G (IgG) level.

**Results:**

OL could relieve renal pathological injury and decrease the abnormal levels of kidney index, serum Cr, CK, BUN, and MDA, as well as increase the immune organ indices and the levels of IgG, GSH, and SOD. Treatment with a high dose of OL had more positive therapeutic effects on ARF than using a low dose of OL.

**Conclusion:**

OL could ameliorate renal dysfunction in glycerol-induced ARF in mice by inhibiting oxidative stress and enhancing immune response.

## 1. Introduction

Acute renal failure (ARF) is a kind of acute urinary dysfunction of kidneys caused by various reasons in a short term, which usually leads to a serious disorder of the body's internal environment. ARF was characterized by acute elevations of serum creatinine (Cr) and blood urea nitrogen (BUN) in hours to days or weeks [1]. ARF has been widely concerned by the medical profession due to its complex pathogenesis and high mortality. Currently, early treatment of ARF focuses on treating the cause and correcting the imbalance of electrolyte and diuresis. Although these treatments can alleviate ARF to some extent, their therapeutic effect is not stable and durable, thus motivating medical researchers to explore new safe and effective medication.

Medicinal fungus in China is considered as one important category of traditional Chinese herbs. Increasing evidence indicated that these fungi and their bioactive ingredients had been screened for antitumor, antivirus, antibacteria, and antithrombosis, and had been helping digestion, lowering blood pressure and sugar, relieving cough and asthma, nourishing the lung and kidney, regulating immunity and metabolism, and so on [2–9].

*Ophiocordyceps sinensis* (named *Cordyceps sinensis* before), commonly known as the Chinese caterpillar fungus [10], is the prime example of medicinal fungi, which has been widely used in traditional Chinese medicine for the treatments of renal failure, bronchitis, pneumonia, and asthma [11]. Clinical studies have shown that *O. sinensis* could cure or relieve several kidney diseases, but the mechanisms remained unclear [9–11]. The nephroprotective (acute and chronic) activity of *O. sinensis* may work through modulating the immune system and ameliorating renal functions and renal oxidative stress [12, 13].

Because of excessive collection and use, the wild resource of *O. sinensis* is decreasing rapidly and the large-scale artificial culture of *O. sinensis* is very hard. *Ophiocordyceps lanpingensis* (OL) has been identified as a new species of *Ophiocordyceps* genus, which belongs to the same genus of *O. sinensis* and they are close relatives [14]. *O. lanpingensis* has been used as an efficient herb treating the disease of urinary systems by the local ethnic people for a long time. Our previous study showed that the chemical composition of *O. lanpingensis* was similar to those of *O. sinensis*. Furthermore, *O. lanpingensis* is easy to be cultured artificially. Thus, it has the potential to be the alternative of *O. sinensis.*

In the present study, based on an ARF mouse model, the effects of OL on ARF were observed systematically using biochemical, immunological, and histopathological indicators. This study will contribute to better understand the mechanism of treating ARF by *Ophiocordyceps* medicinal fungi.

## 2. Materials and Methods

### 2.1. Animals and Grouping

Male mice with C57BL/6 background (6- to 8-week old; 20–25 g body weight) were obtained from Liaoning Changsheng Biotechnology Co. Ltd, China. The mice were maintained in a pathogen-free mouse facility; and clean food and water were supplied with free access. All experiments were performed according to the guidelines for the care of laboratory animals and were proved by the Ethics Committee Guide of Kunming University of Science and Technology.

### 2.2. Drugs

*Ophiocordyceps lanpingensis* (OL) powder was provided by Yunnan Yunbaicao Biotechnology Co. Ltd. which was suspended in 0.25% carboxymethyl cellulose sodium (CMC).

### 2.3. Administration

Mice were randomly divided into four groups, each comprising of 10 animals. The animals were allowed free access to food but deprived of drinking water for 24 hours before glycerol injection. Group 1 serves as normal control group. The animals were treated with saline (10.0 mL/kg/d, intragastric [i.g.]) for 7 days, deprived of drinking water for 24 hours on the sixth day, then were given saline (10.0 mL/kg intramuscular [i.m.]), divided equally among the hind legs. Group 2 is ARF model group. The animals were treated with saline (10.0 mL/kg/d, i.g.) for 7 days, deprived of drinking water for 24 hours on the sixth day, then were given 50% glycerol (10.0 mL/kg, i.m.), divided equally among the hind legs. Group 3 is low-dose OL-treated group. The animals were treated with OL (1.0 g/kg/d, i.g.) for 7 days, deprived of drinking water for 24 hours on the sixth day, then were given 50% glycerol (10.0 mL/kg, i.m.), divided equally among the hind legs. Group 4 is high-dose OL-treated group. The animals were treated with OL (2.0 g/kg/d, i.g.) for 7 days, deprived of drinking water for 24 hours on the sixth day, then were given 50% glycerol (10.0 mL/kg, i.m.), divided equally among the hind legs. The animals were allowed free access to food and water after the glycerol injection for 24 hours [15]. At the end of the treatment, animals were euthanized by CO_2_. The blood was obtained and centrifuged (4000 ×g for 10 min at 4°C) to get serum which was then stored at −80°C until assay. The kidneys, thymus, and spleen were harvested and weighed. The left kidney was frozen at −80°C for subsequent evaluation, while the right kidney was fixed in 4% paraformaldehyde solution for histological sectioning.

### 2.4. Renal Coefficient and Immune Organ Indices

The weight of the mice was measured before death. Renal tissues, thymus tissue, and spleen tissue were collected from mice, washed by normal saline solution (0.9%), and then blotted them with paper. Renal index, thymus index, and spleen index were used to help evaluate renal and immune status.

(1) $$\begin{array}{c} Renal\;index\left(mg/g\right)=\frac{Renal\;weight}{Mice\;weight},\\ Thymus\;index\left(mg/g\right)=\frac{Thymus\;weight}{Mice\;weight},\\ Spleen\;index\left(mg/g\right)=\frac{Spleen\;weight}{Mice\;weight}. \end{array}$$

### 2.5. Serum Biochemical Analysis

The level of serum IgG was detected using immunoglobulin G assay kit (Nanjing Jiancheng Bioengineering Institute, China) in the form of immunoturbidimetric assay. Serum biochemical parameters of BUN and serum Cr levels were measured using urea assay kit (Nanjing Jiancheng Bioengineering Institute, China) and creatinine assay kit (Nanjing Jiancheng Bioengineering Institute, China) in the form of urease method and picric acid colorimetric method, respectively. The activity of serum CK was detected using creatine kinase assay kit (Nanjing Jiancheng Bioengineering Institute, China) in the form of a colorimetric method.

### 2.6. Antioxidant Indices

Kidneys were homogenized in iced saline (0.9% sodium chloride). The homogenates were centrifuged at 800 ×g for 5 minutes at 4°C to separate the nuclear debris. The supernatant obtained was centrifuged at 10,500 ×g for 20 minutes at 4°C to get the postmitochondrial supernatant which was used to assay glutathione (GSH), malondialdehyde (MDA), and superoxide dismutase (SOD) levels. SOD activity was assayed in the form of hydroxylamine method by using SOD assay kit (Nanjing Jiancheng Bioengineering Institute, China), while GSH and MDA levels were assayed in the form of microplate test and thiobarbituric acid (TBA) method by using GSH assay kit (Nanjing Jiancheng Bioengineering Institute, China) and MDA assay kit (Nanjing Jiancheng Bioengineering Institute, China), respectively.

### 2.7. Renal Histopathology

Kidney tissues were embedded in paraffin and used for histopathological examination. Four-micrometer-thick sections were cut, deparaffinized, and hydrated. For light microscopic purpose, paraffin sections were stained with hematoxylin and eosin (H&E). The intact glomeruli, hemorrhage, capillary congestion, and vacuolization of the medullary tubular cells were evaluated.

### 2.8. Statistical Analysis

The results were reported as the mean ± SEM. All of the data were compared by one-way analysis of variance test (ANOVA) while Tukey's multiple comparison test was used to detect significance between all groups. For analysis, a *P* < 0.05 was considered statistical significance. Statistical analysis was performed using SPSS® v.17.0 software.

## 3. Results

### 3.1. Evaluation of a Mouse Model of ARF

**Figure 1:**
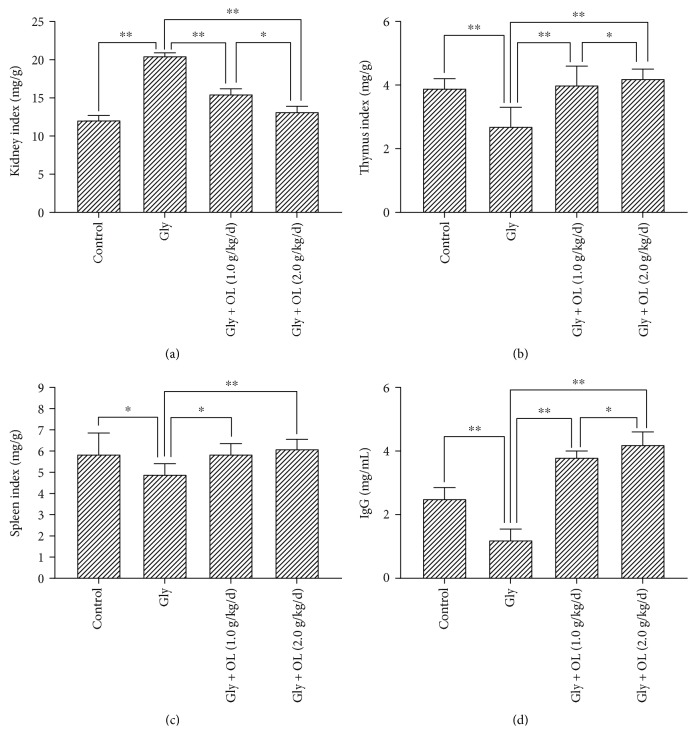
Kidney index, thymus index, spleen index, and serum IgG level. (a) The changes of kidney index in different groups. (b) The changes of thymus index in different groups. (c) The changes of spleen index in different groups. (d) The changes of serum IgG content in different groups. Notes: the statistical significance between the OL-treated groups, normal control group, and acute renal failure (ARF) model group was determined using *Tukey's test*. ^∗^*P* < 0.05 and ^∗∗^*P* < 0.01. Gly: ARF induced by glycerol; OL: *Ophiocordyceps lanpingensis*; IgG: immunoglobulin G.

**Figure 2:**
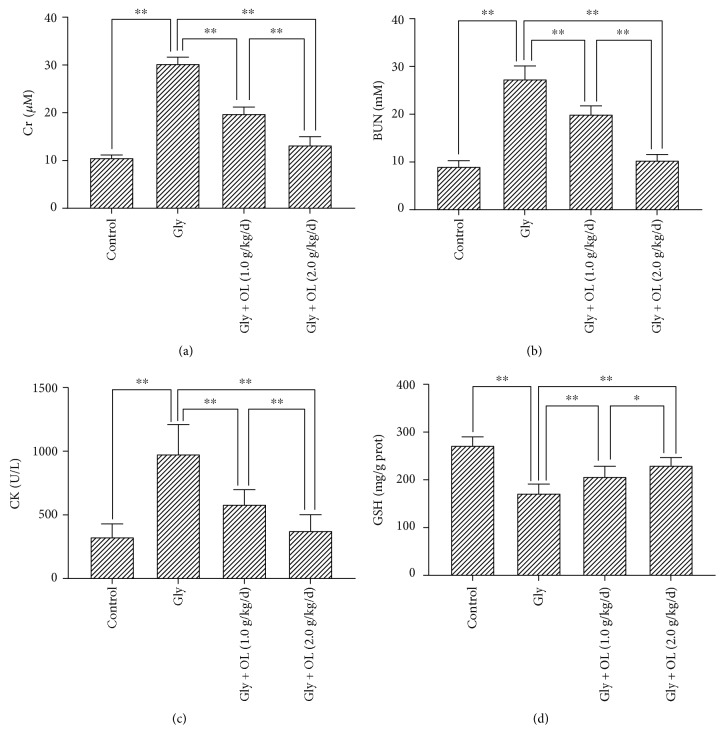
Serum Cr and BUN levels, serum CK activity and kidney tissue GSH level. (a) The effect of OL on glycerol-induced changes in serum Cr. (b) The effect of OL on glycerol-induced changes in BUN. (c) The effect of OL on glycerol-induced changes in serum CK. (d) The effect of OL on glycerol-induced changes in kidney tissue GSH. Notes: the statistical significance between the treated groups, normal control group, and acute renal failure (ARF) model group was determined using *Tukey's test*. ^∗^*P* < 0.05 and ^∗∗^*P* < 0.01. OL: *Ophiocordyceps lanpingensis*; Gly: ARF induced by glycerol; Cr: creatinine; BUN: blood urea nitrogen; CK: creatine kinase; GSH: glutathione.

**Figure 3:**
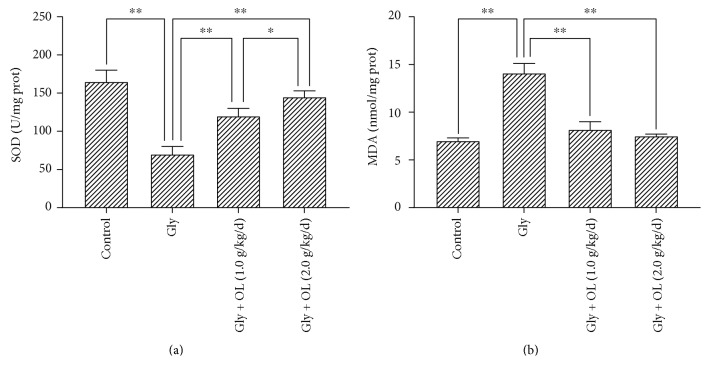
Kidney tissue SOD activity and MDA level. (a) The effect of OL on glycerol-induced changes in kidney tissue SOD. (b) The effect of OL on glycerol-induced changes in kidney tissue MDA. Notes: the statistical significance between the treated groups, normal control group, and acute renal failure (ARF) model group was determined using *Tukey's test*. ^∗^*P* < 0.05 and ^∗∗^*P* < 0.01. OL: *Ophiocordyceps lanpingensis*; Gly: ARF induced by glycerol; SOD: superoxide dismutase; MDA: malondialdehyde.

Comparing with the control group, ARF caused by glycerol injection in mice resulted in significant changes in immune organs and IgG. There were statistically significant decreases in thymus index (*P* < 0.01), spleen index (*P* < 0.05), and serum IgG (*P* < 0.01) (Gly group in Figures 1(b), 1(c), and 1(d)). Levels of renal GSH (*P* < 0.01) and SOD (*P* < 0.01) were also significantly reduced (Gly group in Figures 2(d) and 3(a)); meanwhile, the kidney index (*P* < 0.01), levels of serum Cr (*P* < 0.01), serum CK (*P* < 0.01), BUN (*P* < 0.01), and renal MDA (*P* < 0.01) were enhanced much more (Gly group in Figures 1(a), 2(a), 2(b), 2(c), and 3(b)). Such results indicated that ARF induced severe failure in kidney functions and oxidative stress which suggested that the animal model of ARF was gotten definitely and efficiently.

### 3.2. OL Improved Immunity of Mice in Glycerol-Induced ARF

Intragastric administration of OL for 7 days in both doses of 1.0 g/kg/d and 2.0 g/kg/d resulted in significant improvement in immunity compared with ARF model group. A statistically significant increase in thymus index (*P* < 0.01), spleen index (*P* < 0.05), and serum IgG level (*P* < 0.01) were shown in Figures 1(b), 1(c), and 1(d). More efficient enhancement of related immunity parameters was observed in the group which received OL in a dose of 2.0 g/kg/d (Figures 1(b) and 1(d)). Such results indicated that the effects of OL in AFR may depend on the dose.

### 3.3. OL Prevented Damage of Kidney Functions and Improved Oxidative Stress of Kidney in Glycerol-Induced ARF

The serum Cr and BUN were analyzed in this study, which were two important biomarkers of renal function. In addition, chemical- or ischemia-induced renal failure is generally associated with a remarkable increase of MDA level and decreases of GSH and SOD levels. Rhabdomyolysis was monitored by creatine kinase (CK) level, which was a representative symptom caused by glycerol. Treatments of OL in both doses of 1.0 g/kg/d and 2.0 g/kg/d showed significant improvements in kidney functions and oxidative stress compared with ARF model group. There were significant decreases in kidney index (*P* < 0.01), serum Cr (*P* < 0.01), serum CK (*P* < 0.01), BUN (*P* < 0.01), and renal MDA (*P* < 0.01) (Figures 1(a), 2(a), 2(b), 2(c), and 3(b)), whereas enhancements in renal GSH (*P* < 0.01) and SOD (*P* < 0.01) were observed (Figures 2(d) and 3(a)). More prominent improvements in kidney functions and oxidative stress were shown in a dose of 2.0 g/kg/d OL group (Figures 1(a), 2(a), 2(b), 2(c), 2(d), and 3(a)).

### 3.4. OL Administration Caused Regression of Renal Histopathological Changes

**Figure 4:**
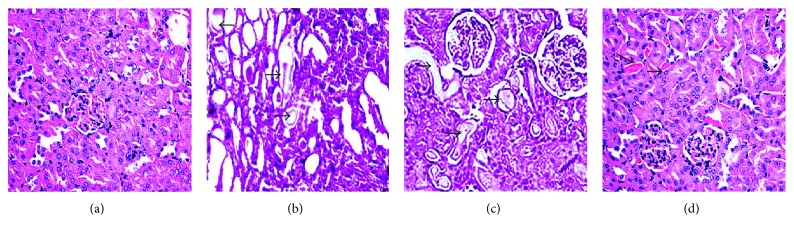
Hematoxylin and eosin results in mice's kidney tissues (magnification ×400). (a) Normal control group. (b) ARF model group. (c) 1.0 g/kg/d OL-treated group. (d) 2.0 g/kg/d OL-treated group. Notes: (a) normal architecture of kidney in the normal healthy group. (b) Many necrotic cortical tubules with casts (arrowheads), tubular dilation, and vacuolation were seen. (c**)** Severe necrotic tubules with some casts (arrowheads) were present. (d) Occasional necrotic tubules with casts (arrowheads) were seen. OL: *Ophiocordyceps lanpingensis*; ARF: acute renal failure.

The horizontal section of mouse kidney had no obvious pathological changes, showing normal structure of kidney tissue and integrality of cells in tubule epithelium in normal control group (Figure 4(a)). In ARF model group, many necrotic tubules with casts, tubular dilation, and vacuolation were seen (Figure 4(b)). Intragastric administration of OL in different doses resulted in significant regression of renal histopathological changes compared with ARF model group, especially when received OL in a dose of 2.0 g/kg/d (Figures 4(c) and 4(d)).

## 4. Discussion

ARF is a common clinical emergency with abrupt loss of kidney function, which may lead to a number of complications and even death. Studies have demonstrated that the pathogenesis of ARF was associated with the oxidative stress and a host of inflammatory mediators and cell-mediated immune responses [15–20]. A glycerol-induced mouse model can simulate ARF which is characterized by a significant increase of Cr, CK, and BUN in serum. CK is the most sensitive damage index for muscle cell damage and marks the occurrence of rhabdomyolysis [15]. In the animal model, significant structural changes of kidney including tubular dilatation, vacuolation, necrosis, and cellular debris could be observed [21–23].

The conventional treatments about ARF include the underlying causes and supportive care; furthermore, treatment with Chinese medicine has been applied widely in clinic. In recent years, herbs and their effective components are considered as promising therapeutic options for ARF and many studies indicated the potential role of them in reducing renal dysfunction after ARF [24–27]. As a famous traditional Chinese herb, the beneficial effects of *O. sinensis* or its water-soluble polysaccharide on various renal diseases have been proven [28]. So far, with the extreme lack of the natural resource of *O. sinensis*, the substitution of *O. sinensis* needs to be studied. *O. lanpingensis* (OL), a Chinese herb similar with *O. sinensis* which could protect against ARF, has been proven to contain bioactive constituents that may have pharmacological effects such as antioxidant, anti-inflammatory, and immune activation. In this study, we explored the protective effects of OL on glycerol-induced ARF in mice and firstly found that OL could enhance immunity, protect kidney functions, and relieve oxidative stress as well as renal pathological damage.

The possible explanation for the benefits in renal function recovery following administration of OL may be due to its role in increasing immunity as well as reducing oxidative stress. Oxidative stress is closely related to human health and plays an important role in the pathogenesis of glycerol-induced ARF. Normally, the production and elimination of oxygen free radicals in the human body are balanced. But when the body's antioxidant system is disordered, excessive oxygen free radicals will be produced; thus, the oxygen free radical metabolism in the body will be imbalanced, leading to cell damage and then even cause heart disease, cancer, or other serious problems [29, 30]. During physiological activities, the body produces reactive oxygen species (ROS) continuously. The biological activity of ROS is very strong, which plays a positive role in cell division, growth, anti-inflammation, and so on. Nevertheless, ROS is the most significant contributing factor to oxidative stress in complex systems and excessive ROS may cause cell aging, body damage, inflammation, immune disorders, and other diseases.

In order to evaluate the antioxidant capacity of the body, the activity of SOD and the contents of renal GSH and MDA in the mice were measured in this study. SOD is one of the main free radical scavengers in the body, which plays an important role in the oxidation and antioxidation balance in the organism. SOD can remove excessive free radicals and reduce the negative effects of free radicals on biofilm and other tissues; meanwhile, GSH is another important free radical scavenger with strong protective effects [31–36]. High or low content of MDA in the tissues indirectly reflects the severity of the cells attacked by free radicals. The current study indicated that oral administration of OL caused significant increases in renal SOD and GSH while decreasing the renal MDA to normal condition compared with their levels in ARF model group. Moreover, the group received OL in a dose of 2.0 g/kg/d representing remarkable effects in all physiological parameters which were closed to those of normal group. The effects of renoprotection were dose dependent.

SOD and MDA are important in tissues and organs for their functions in the body's oxidative stress and immune protection. Correspondingly, the immune response of body can ameliorate oxidative stress and inflammation [37, 38]. Besides oxidative stress, another factor that plays a role in the pathogenesis of nephrotoxicity is the process of immunity. The occurrence of body damage is accompanied by an inflammatory response which regulates multiple physiological metabolisms. Such effects depend on the concentrations of cytokines, chemokines, and other inflammatory molecules. Very low levels of inflammatory molecules are enough to induce immune responses. IgG is one of the critical substances in the immune system of the body, which is synthesized and secreted by plasma cells in spleen and lymph nodes. IgG plays an important role in the immune and physiological adjustment [39, 40]. As the main antibody composition in the serum, IgG is widely distributed in tissues, which possesses anti-infection function. The content of IgG is a crucial detection index of humoral immunity while the immune organ index is an important and intuitive parameter to reflect the immune status as well [17–19].

To explore the effects of immune role of OL in the present study, thymus index, spleen index, and IgG concentration were examined. The results showed that OL could significantly increase thymus index, spleen index, and the level of serum IgG in mice compared with those in the ARF model. When using the dose of 2.0 g/kg/d OL, the thymus and spleen indices in ARF mice were almost recovered to normal (control group), suggesting that the destroyed immune system might be established again.

## 5. Conclusion

In conclusion, this study showed that OL could relieve the renal injury caused by glycerol. OL ameliorated renal dysfunction of ARF by inhibiting oxidative stress and improving the body's immunity. In future studies, we will explore the definite bioactive components in OL and reveal the correlation between biological effects and these components, thus to provide strong evidence for application of OL.

## References

[1] Nissenson A. R. (1998). Acute renal failure: definition and pathogenesis. *Kidney International Supplements*.

[2] Zhao Y. Y., Li H. T., Feng Y. L., Bai X., Lin R. C. (2013). Urinary metabonomic study of the surface layer of *Poria cocos* as an effective treatment for chronic renal injury in rats. *Journal of Ethnopharmacology*.

[3] Joob B., Wiwanitkit V. (2015). Linzhi (*Ganoderma lucidum*); evidence of its clinical usefulness in renal diseases. *Journal of Nephropharmacology*.

[4] Zhong D., Wang H., Liu M. (2015). *Ganoderma lucidum* polysaccharide peptide prevents renal ischemia reperfusion injury via counteracting oxidative stress. *Scientific Reports*.

[5] Zhao Y. Y., Feng Y. L., Bai X., Tan X. J., Lin R. C., Mei Q. (2013). Ultra performance liquid chromatography-based metabonomic study of therapeutic effect of the surface layer of *Poria cocos* on adenine-induced chronic kidney disease provides new insight into anti-fibrosis mechanism. *PLoS One*.

[6] Yu S. H., Dubey N. K., Li W. S. (2016). *Cordyceps militaris* treatment preserves renal function in type 2 diabetic nephropathy mice. *PLoS One*.

[7] Song J., Wang Y., Liu C. (2016). *Cordyceps militaris* fruit body extract ameliorates membranous glomerulonephritis by attenuating oxidative stress and renal inflammation via the NF-κB pathway. *Food and Function*.

[8] Du F., Li S., Wang T. (2015). *Cordyceps sinensis* attenuates renal fibrosis and suppresses BAG3 induction in obstructed rat kidney. *American Journal of Translational Research*.

[9] Chiu C. H., Chyau C. C., Chen C. C., Lin C. H., Cheng C. H., Mong M. C. (2014). Polysaccharide extract of *Cordyceps sobolifera* attenuates renal injury in endotoxemic rats. *Food and Chemical Toxicology*.

[10] Sung G. H., Hywel-Jones N. L., Sung J. M., Luangsa-Ard J. J., Shrestha B., Spatafora J. W. (2007). Phylogenetic classification of *Cordyceps* and the clavicipitaceous fungi. *Studies in Mycology*.

[11] Zhu J. S., Halpern G. M., Jones K. (1998). The scientific rediscovery of an ancient Chinese herbal medicine: *Cordyceps sinensis* : part I. *Journal of Alternative and Complementary Medicine*.

[12] Hua K. F., Hsu H. Y., Chao L. K. (2007). Ganoderma lucidum polysaccharides enhance CD14 endocytosis of LPS and promote TLR4 signal transduction of cytokine expression. *Journal of Cellular Physiology*.

[13] Lin J., Chang Y. J., Yang W. B., Alice L. Y., Wong C. H. (2014). The multifaceted effects of polysaccharides isolated from *Dendrobium huoshanense* on immune functions with the induction of interleukin-1 receptor antagonist (IL-1 ra) in monocytes. *PLoS One*.

[14] Chen Z. H., Dai Y. D., Yu H. (2013). Systematic analyses of *Ophiocordyceps lanpingensis* sp. nov., a new species of *Ophiocordyceps* in China. *Microbiological Research*.

[15] Homsi E., Janino P., Faria J. B. (2006). Role of caspases on cell death, inflammation, and cell cycle in glycerol-induced acute renal failure. *Kidney International*.

[16] N. Bank, Aynedjian H. S. (1992). Role of thromboxane in impaired renal vasodilatation response to acetylcholine in hypercholesterolemic rats. *Journal of Clinical Investigation*.

[17] Unis A. (2014). Açai berry extract attenuates glycerol-induced acute renal failure in rats. *Renal Failure*.

[18] Singh A. P., Muthuraman A., Jaggi A. S. (2012). Animal models of acute renal failure. *Pharmacological Reports*.

[19] Asmari A. K. A., Sadoon K. T. A., Obaid A. A., Yesunayagam D., Tariq M. (2017). Protective effect of quinacrine against glycerol-induced acute kidney injury in rats. *BMC Nephrology*.

[20] Nishida K., Watanabe H., Ogaki S. (2015). Renoprotective effect of long acting thioredoxin by modulating oxidative stress and macrophage migration inhibitory factor against rhabdomyolysis-associated acute kidney injury. *Scientific Reports*.

[21] Gu H. X., Yang M., Zhao X. M., Zhao B., Sun X., Gao X. (2014). Pretreatment with hydrogen-rich saline reduces the damage caused by glycerol-induced rhabdomyolysis and acute kidney injury in rats. *Journal of Surgical Research*.

[22] Liano F., Junco E., Pascual J. (1998). The spectrum of acute renal failure in the intensive care unit compared with that seen in other setting. *Kidney International Supplements*.

[23] Cardinal J. S., Zhan J. H., Wang Y. N. (2010). Oral hydrogen water prevents chronic allograft nephropathy in rats. *Kidney International*.

[24] Ustundag S., Sen S., Yalcin O., Ciftci S., Demirkan B., Ture M. (2009). L-Carnitine ameliorates glycerol-induced myoglobinuric acute renal failure in rats. *Renal Failure*.

[25] Bowmer C. J., Collis M. G., Yates M. S. (1986). Effect of the adenosine antagonist 8-phenyltheophylline on glycerol-induced acute renal failure in the rats. *British Journal of Pharmacology*.

[26] Zhou J., Zhang H. A., Lin Y. (2014). Protective effect of ginsenoside against acute renal failure via reduction of renal oxidative stress and enhanced expression of ChAT in the proximal convoluted tubule and ERK1/2 in the paraventricular nuclei. *Physiology Research*.

[27] Lee Y. K., Chin Y. W., Choi Y. H. (2013). Effects of Korean red ginseng extract on acute renal failure induced by gentamicin and pharmacokinetic changes by metformin in rats. *Food and Chemical Toxicology*.

[28] Jin X., Ying H., Chen X. X., Zheng S.-C., Chen P., Mo M.-H. (2016). The mechanisms of pharmacological activities of *Ophiocordyceps sinensis* fungi. *Phytotherapy Research*.

[29] Kang D. G., Oh H., Sohn E. J. (2004). Lithospermic acid B isolated from *Salvia miltiorrhiza* ameliorates ischemia/reperfusion-induced renal injury in rats. *Life Science*.

[30] Chan K. W. K., Ho W. S. (2015). Anti-oxidative and hepatoprotective effects of lithospermic acid against carbon tetrachloride-induced liver oxidative damage in vitro and in vivo. *Oncology Reports*.

[31] Jia L. F., Yang Y., Li Q. Y. (2012). Antioxidant effect of glycoprotein from *Taraxacum mongolicum in votro* and *in vivo*. *Acta Botanica Boreali-Occidentalia Sinica*.

[32] Yoon W. S., Chae Y. S., Hong J., Park Y. K. (2011). Antitumor therapeutic effects of a genetically engineered Salmonella typhimurium harboring TNF-α in mice. *Applied Microbiology and Biotechnology*.

[33] Zhong J. C., Zhang Y., Ding Z. T., Ke Y. (2011). Effect of polysaccharide extract from artificial *Cordyceps sinensis* on immune function of mouse. *Acta Scientiarum Naturalium Universitatis Sunyatseni*.

[34] Wang F., Zhang C. J., Wang L. (2008). Empirical study progress about immune modulatory effect of extractive of artificial cultured *Cordyceps sinensis*. *Medical Recapitulate*.

[35] Manikandan R., Beulaja M., Thiagaraja R. (2004). Ameliorative effect of ferulic acid against renal injuries mediated by nuclear factor-kappaB during glycerol-induced nephrotoxicity in Wistar rats. *Renal Failure*.

[36] Vlahovic P., Cvetkovic T., Savic V., Stefanović V. (2007). Dietary curcumin does not protect kidney in glycerol induced acute failure. *Food and Chemical Toxicology*.

[37] Inoue T. (2017). M1 macrophage triggered by Mincle leads to a deterioration of acute kidney injury. *Kidney International*.

[38] Lv L. L., Tang P. M., Li C. J. (2017). The pattern recognition receptor, Mincle, is essential for maintaining the M1 macrophage phenotype in acute renal inflammation. *Kidney International*.

[39] Cicciarelli J. C., Lemp N. A., Chang Y. (2017). Renal transplant patients biopsied for cause and tested for C4d, DSA, and IgG subclasses and C1q: which humoral markers improve diagnosis and outcomes?. *Journal of Immunology Research*.

[40] Kurtenkov O., Klaamas K. (2017). Hidden IgG antibodies to the tumor-associated Thomsen-Friedenreich antigen in gastric cancer patients: lectin reactivity, avidity, and clinical relevance. *BioMed Research International*.

